# Upfront F18-choline PET/CT versus Tc99m-sestaMIBI SPECT/CT guided surgery in primary hyperparathyroidism: the randomized phase III diagnostic trial APACH2

**DOI:** 10.1186/s12902-020-00667-5

**Published:** 2021-01-07

**Authors:** Elske Quak, Audrey Lasne Cardon, Renaud Ciappuccini, Charline Lasnon, Vianney Bastit, Véronique Le Henaff, Barbara Lireux, Gauthier Foucras, Cyril Jaudet, Celia Berchi, Jean-Michel Grellard, Justine Lequesne, Bénédicte Clarisse, Stéphane Bardet

**Affiliations:** 1 Department of Nuclear Medicine and Thyroid Unit, Centre François Baclesse, Avenue Général Harris, F-14000, 14076 Caen cedex 5, France; 2 Department of Head & Neck Surgery, Centre François Baclesse, F-14000 Caen, France; 3 INSERM 1086 ANTICIPE, F-14000 Caen, France; 4 Normandie Univ, UNICAEN, Caen, France; 5 Clinical Research Department, Centre François Baclesse, F-14000 Caen, France

**Keywords:** Parathyroid adenoma, Primary hyperparathyroidism, MIBI SPECT/CT, F18-choline PET/CT, Minimally invasive surgery, Medico-economic evaluation

## Abstract

**Background:**

The common endocrine disorder primary hyperparathyroidism (PHPT) can be cured by surgery. Preoperative localization of parathyroid adenoma (PTA) by imaging is a prerequisite for outpatient minimally invasive parathyroidectomy (MIP). Compared to inpatient bilateral cervical exploration (BCE) which is performed if imaging is inconclusive, MIP is superior in terms of cure and complication rates and less costly. The imaging procedure F18-choline (FCH) PET/CT outperforms Tc99m-sestaMIBI (MIBI) SPECT/CT for PTA localization, but it is much costlier. The aim of this study is to identify the most efficient first-line imaging modality for optimal patient care in PHPT without added cost to society.

**Methods:**

We will conduct a multicenter open diagnostic intervention randomized phase III trial comparing two diagnostic strategies in patients with PHPT: upfront FCH PET/CT versus MIBI SPECT/CT. The primary endpoint is the proportion of patients in whom the first-line imaging method results in successful MIP and cure. Follow-up including biological tests will be performed 1 and 6 months after surgery. The main secondary endpoint is the social cost of both strategies. Other secondary endpoints are as follows: FCH PET/CT and MIBI SPECT/CT diagnostic performance, performance of surgical procedure and complication rate, FCH PET/CT inter- and intra-observer variability and optimization of FCH PET/CT procedure. Fifty-eight patients will be enrolled and randomized 1:1.

**Discussion:**

FCH PET/CT is a highly efficient but expensive imaging test for preoperative PTA localization and costs three to four times more than MIBI SPECT/CT. Whether FCH PET/CT improves patient outcomes compared to the reference standard MIBI SPECT/CT is unknown. To justify its added cost, FCH PET/CT-guided parathyroid surgery should lead to improved patient management, resulting in higher cure rates and fewer BCEs and surgical complications.

In the previous phase II APACH1 study, we showed that second-line FCH PET/CT led to a cure in 88% of patients with negative or inconclusive MIBI SPECT/CT. BCE could be avoided in 75% of patients and surgical complication rates were low. We therefore hypothesize that upfront FCH PET/CT would improve patient care in PHPT and that the reduction in clinical costs would offset the increase in imaging costs.

**Trial registration:**

NCT04040946, registered August 1, 2019.

**Protocol version**

Version 2.1 dated from 2020/04/23.

## Background

Primary hyperthyroidism (PHPT) is a common endocrine disorder characterized by hypercalcemia and elevated or inappropriately normal parathyroid hormone (PTH) levels. Autonomous PTH production by a single parathyroid adenoma (PTA) situated behind or just underneath the thyroid is the most frequent cause [1]. PHPT can be cured by surgical removal of the PTA. With the advent of preoperative PTA localization by medical imaging, conventional inpatient bilateral cervical exploration (BCE) is tending to be replaced by minimally invasive surgery (MIP). MIP has superior cure and complication rates compared to BCE and is less costly [2].

One of the most frequently used 3D imaging techniques for PTA localization is parathyroid scintigraphy including SPECT/CT acquisition. Parathyroid SPECT/CT is a readily available, non-invasive, and slightly irradiating imaging method. Several parathyroid scintigraphy protocols exist and most of the performance data come from retrospective studies. Reported detection rates and sensitivities vary widely [3–9]. In our population, parathyroid SPECT/CT with the tracer Tc99m-sestaMIBI (MIBI) led to PTA detection in about two-thirds of patients, meaning that BCE or active surveillance was proposed to one-third of patients [10].

Recent studies and the results of our phase II study APACH1 concordantly suggest the superiority of F18-choline (FCH) PET/CT for PTA localization [5, 11–13]. In the APACH1 study [12], 25 patients with PHPT and negative or inconclusive MIBI SPECT/CT results underwent FCH PET/CT, resulting in 22 FCH PET/CT guided surgeries: 17 MIPs, 1 bilateral cervical exploration for multifocality and 4 other surgical procedures. BCE could be avoided in 75% of patients. Sensitivity and positive predictive value (PPV) were 91 and 87%, respectively. Operative time was halved (44 versus 100 min), the surgical complication rate was low, and the short-term cure rate was 88%. Exposure to ionizing radiation due to FCH PET/CT was low. A recent prospective diagnostic cohort study in 100 patients with PHPT comparing FCH PET/CT to MIBI SPECT/CT showed the superior accuracy of FCH PET/CT, especially for the detection and correct localization of small PTAs [5].

For several reasons, FCH PET/CT currently has no place as a first-line imaging modality in the preoperative workup of PHPT patients: firstly, FCH has no marketing authorization for parathyroid imaging; secondly, FCH PET/CT is about three to four times costlier for society than MIBI SPECT/CT; and lastly FCH PET/CT is slightly less available. However, because of the high PTA detection rate and superior diagnostic performance of FCH PET/CT, the question arises whether FCH PET/CT should be performed as a first-line imaging technique instead of MIBI SPECT/CT, and whether this approach could be cost-effective. In this context, we propose to conduct a diagnostic intervention randomized phase III study aiming to evaluate the most efficient first-line imaging modality for optimal patient care in PHPT without added cost to society.

## Methods/design

### Trial objectives

#### Primary objective

The main objective is to compare for each diagnostic strategy the proportion of patients in which the first-line imaging modality (FCH PET/CT or MIBI SPECT/CT) results in successful surgery and cure. Successful surgery and cure are defined as the decision to perform a true positive MIP combined with cure of the patient defined as normalization of serum calcium levels 1 month after surgery.

#### Secondary objectives

The secondary objectives are as follows:

- To perform a medico-economic analysis of the social costs of each diagnostic strategy by considering the mean cost of management per patient.
- To evaluate the diagnostic performance (sensitivity, specificity, positive and negative likelihood rates) of each diagnostic strategy.
- For each diagnostic strategy, to assess the number of unsuccessful surgical procedures (disregarding the surgical procedure type) defined by the persistence of PHPT 6 months after surgery.
- To estimate the number of surgical complications, i.e. infections, hematomas, and lesions of the recurrent laryngeal nerve.
- To assess the inter- and intra-observer variability of FCH PET/CT and MIBI SPECT/CT interpretation, using Cohen’s kappa coefficients between two independent observers, for the first and second (3 months later) interpretations, as well as between the first and second interpretations for each of the two observers.
- To explore the relationship between the positivity of imaging exams and baseline serum PTH level.
- To assess the performance of FCH PET/CT by comparing early PET acquisition at 10 min versus 60 min after injection to detect PTA (sensitivity and semi-quantitative analysis).
- To assess patients’ satisfaction of the perioperative period by scoring the six dimensions (attention, privacy, information, pain, discomfort, and waiting times) of the 26-item EVAN-G self-questionnaire administered 4 to 48 h post-surgery and before the end of hospitalization.

### Study population

Eligibility criteria are detailed in Table 1. Briefly, eligible patients have a biologically confirmed PHPT for which a parathyroidectomy is required according to the international guidelines [14].

**Table 1:** Study eligibility criteria

**Inclusion criteria**	- Age 18 years or older - Patients with PHPT needing parathyroidectomy - Biological tests confirming PHPT diagnosis (elevated serum PTH and calcium levels) - Negative pregnancy test upon inclusion in women of childbearing age - Patients with health care insurance - Signed informed consent
**Exclusion criteria**	- Known allergy for MIBI or FCH or one of its excipients - Pregnant or breastfeeding women - Previous history of parathyroid surgery - Patients with multiple endocrine neoplasia type 1 (MEN1) - Any medical conditions or associated psychopathology that may compromise patient’s ability to participate in study - Patient deprived of freedom or under guardianship.

### Trial design

**Fig. 1:**
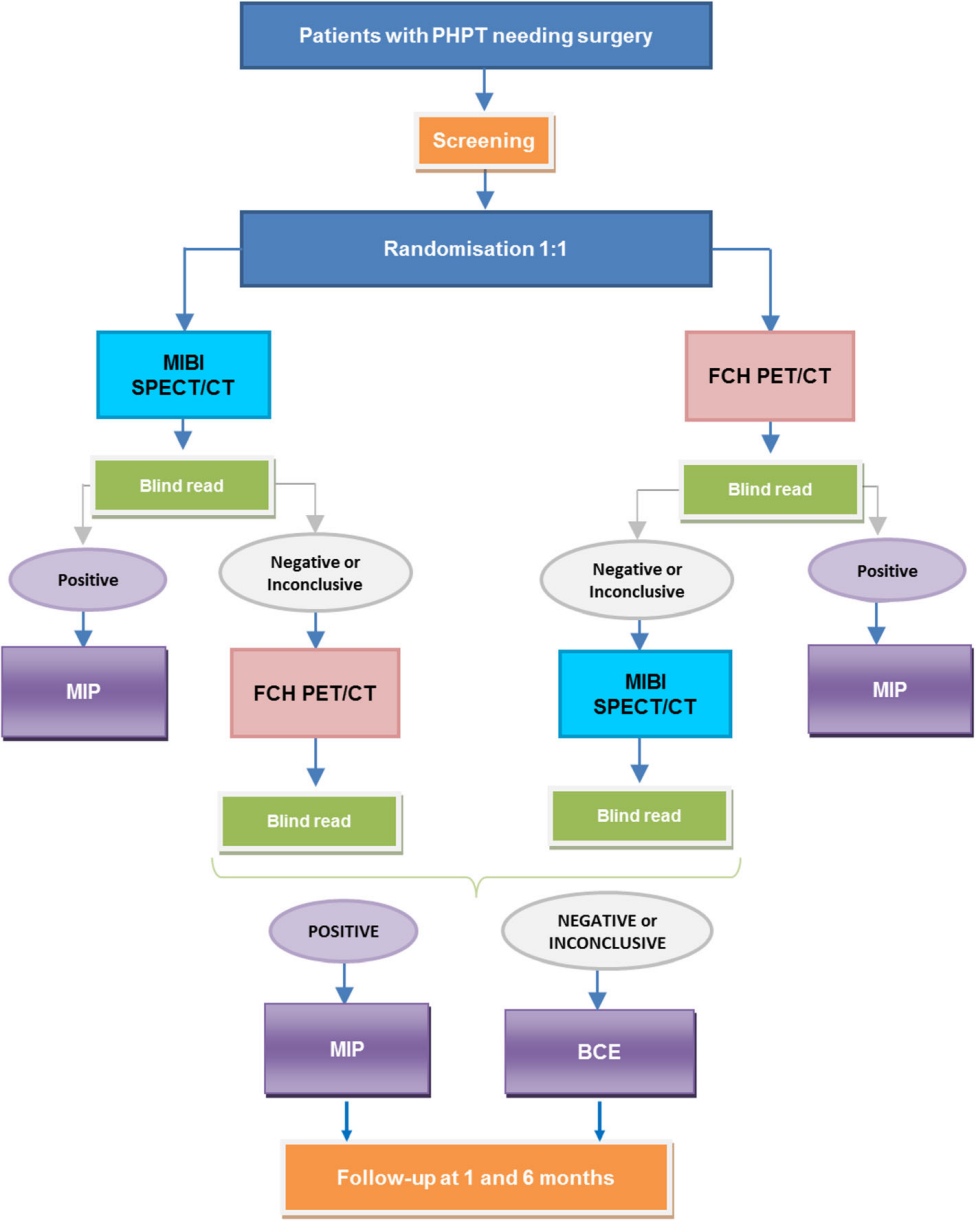
APACH2 study design

The study protocol and this manuscript have been written in accordance with standard protocol items, namely recommendations for interventional trials (SPIRIT). The APACH2 study is a multicenter open diagnostic intervention randomized phase III trial comparing two diagnostic strategies: upfront FCH PET/CT versus MIBI SPECT/CT for preoperative PTA localization in PHPT patients. The flowchart of the study can be found in Fig. 1.

### Study sites

The list of study sites is available on https://clinicaltrials.gov/ct2/show/NCT04040946.

### Trial procedures and assessments

The overview of study procedures and assessments can be found in Table 2. The study will be proposed by investigators to eligible patients.

**Table 2:** Overview of study assessments of APACH2 trial

	Screening *(before inclusion)*	Before surgery	Surgery	Follow-up	
Month (M)				M 1	M 6
Informed consent	✓				
Medical and demographic history	✓				
Physical examination (weight, length)	✓				
Pregnancy (urinary or blood) test	✓ *(within 8 days)*				
Neck ultrasound	✓				
Blood tests^a^	✓			✓	✓
FCH PET/CT and/or MIBI SPECT/CT		✓			
Adverse events		✓	✓	✓	✓
PHPT-related health care consumption		✓	✓	✓	✓

^a^serum values of calcium, parathyroid hormone (PTH), albumin, phosphorus, vitamin D and creatinine, creatinine clearance calculated according to MDRD formula

### Baseline evaluations

Upon inclusion, eligible patients with signed informed consent will undergo a clinical exam (assessing relevant antecedents, PHPT history and current concomitant treatments) and a standard neck ultrasound before computer-generated randomization. As for biological examinations, serum calcium, parathyroid hormone (PTH), albumin, phosphorus, vitamin D and creatinine will be measured for all patients, and creatinine clearance will be calculated according to the MDRD formula. Pregnancy test will be performed 8 days before imaging in women of childbearing age.

### Imaging protocols

FCH PET/CT and/or MIBI SPECT/CT will be performed within 12 weeks before parathyroid surgery. A minimum of 48 h will be respected between both imaging exams.

#### FCH pet/CT

Patients will fast for 4 h. Ten and 60 min after intravenous injection of 1.5 MBq/kg of FCH, a low-dose CT will be performed, followed by a two-bed position PET acquisition of 10 min covering the neck and upper chest in 3D list-mode. The injected activity and the exact delay between injection and the start of acquisition will be noted. Image analysis will be performed on dedicated workstations. Only the 60-min delay images will be used for the clinical FCH PET/CT report. The 10-min delay images will be used for the ancillary study (see further).

#### MIBI SPECT/CT

MIBI SPECT/CT will be performed on Siemens SPECT/CT systems (Siemens Medical Solutions). After intravenous injection of 740 MBq of MIBI, an early pinhole acquisition of the anterior lower neck will be performed 10 min after injection, followed by a SPECT/CT acquisition of the neck and upper chest 90 min after injection. Image analysis will be performed on dedicated workstations. A detailed description of the MIBI SPECT/CT protocol can be found elsewhere [10].

#### Image interpretation

Each FCH PET/CT or MIBI SPECT/CT exam will be interpreted by two experienced nuclear medicine physicians in a blinded fashion. The result will be considered positive in the event of clear focal uptake(s) in a predisposing area. The exact location of each focus will be noted (the side and upper or lower position, or the ectopic position), and when measurable the maximum transverse CT diameter. A negative result is defined as the absence of focal uptake. An inconclusive result is defined as faint uptake compared to the surrounding background without CT substrate or uptake most likely related to a thyroid nodule. A concordant result between the two interpreters will be directly communicated to the surgeon. In the event of discordance, a third read will be performed at the interdisciplinary meeting and communicated to the surgeon. A second blind read of all exams will be performed 3 months after the last inclusion by the same readers in order to estimate the intra-observer agreement.

### Surgery

All patients will undergo surgery within 12 weeks following imaging by a dedicated head and neck surgeon. In the event of positive imaging, an outpatient MIP will be performed. The surgical procedure will be adapted in the event of suspected multiple or ectopic PTAs. If imaging is negative or inconclusive, a conventional inpatient BCE will be performed. The exact location of each resected specimen will be noted, together with the total surgery time and surgical complications, if any. Vocal cord function will be tested by laryngoscopy before discharge. Patients’ satisfaction with the perioperative period will be assessed using the EVAN-G self-questionnaire administered within 48 h after surgery [15].

### Histology

During surgery, an intra-operative frozen section will be performed to confirm the presence of parathyroid tissue. Final analysis will be performed on paraffin-wax embedded sections stained with hematoxylin and eosin. If required, immunohistochemistry with anti-PTH antibody will be performed. Parathyroid adenoma and parathyroid hyperplasia will be considered true positive.

### Follow-up

Clinical and biological assessments will be performed 1 and 6 months post-surgery. All health care consumption related to PHPT care during the follow-up period will be noted.

### Ancillary imaging study

The protocol includes one ancillary study concerning the FCH PET/CT procedure. In the current literature, time frames between FCH injection and PET acquisition vary from 5 to 120 min. As the biokinetics of FCH are rapid [16], a short time frame seems feasible, thereby shortening the length of stay of the patient in the nuclear medicine department. Therefore, we will compare the diagnostic performance at 10 and 60 min.

### Statistical overview

#### Sample size calculation

Since the proportion of patients oriented towards the appropriate surgical strategy according to first-line imaging is 90% with FCH PET/CT [12] and 60% with MIBI SPECT/CT [8, 10], 50 evaluable patients (25 per arm) will be necessary to detect such a difference in proportions with an alpha risk of 5% and a power of 80% (unilateral test). We planned to include 58 patients to anticipate 15% of possible non-assessable patients. Based on the results of our published APACH1 study [12] where a sensitivity of around 90% was observed with FCH PET/CT, we believe that a unilateral test is sufficient in the present design to demonstrate the superiority of FCH PET/CT compared to MIBI SPECT/CT.

#### Statistical analyses

For the main objective, the proportions of patients oriented to the appropriate surgical strategy according to first-line imaging (FCH PET/CT or MIBI SPECT/CT) will be compared using a Chi^2^ test with a unilateral alpha risk of 5%. We will also calculate Cohen’s kappa coefficients (with their 95% confidence interval), which will be considered strong if greater than 0.60, as quasi-perfect if greater than 0.80, as perfect if equal to 1 and as unsatisfactory otherwise. The performances (sensitivity, specificity, error rate, positive and negative likelihood ratios) of the two interventions (FCH PET/CT or MIBI SPECT/CT) will be evaluated using univariate analyses, Chi^2^ test or Fisher’s exact nonparametric test. It is also planned to use multivariate logistic regression models to identify other possible parameters associated with the correct surgical strategy. For patients who have benefited from the two imaging techniques sequentially, we will report their socio-demographic and clinical characteristics, as well as the proportion of minimally invasive surgeries and bilateral explorations.

### Economic evaluation

The cost-effectiveness analysis will be conducted from the French collective perspective. In this context, only direct hospital costs will be considered in the analysis. They will include the costs related to diagnostic procedures, surgery (operative procedure, duration of intervention and hospital stay, anesthesia) as well as costs of potential complications, i.e. infections, hematomas, lesions of the recurrent laryngeal nerve, chronic hypoparathyroidism or persistent hyperparathyroidism.

The efficacy criterion will be the rate of cure 1 month after surgery, defined as normalization of serum calcium levels at 1 month. A differential cost-efficacy ratio will be estimated to establish the mean cost required to cure one additional patient while switching from the standard diagnostic strategy to the new one. A sensitivity analysis on uncertain model parameters will be performed to assess the robustness of the cost-efficacy ratio.

### Data management

A Web-Based Data Capture (WBDC) system will be used for randomization, data collection and query handling. The investigator will ensure that data are recorded on the eCRFs as specified in the trial protocol and in accordance with the instructions provided. The investigator ensures the accuracy, completeness, and timeliness of the data recorded as well as of the provision of answers to data queries according to the Clinical Study Agreement. The investigator will be in charge of signature of the completed eCRFs. A copy of the completed eCRFs will be archived at the study site.

## Discussion

Current literature suggests the superior diagnostic accuracy of FCH PET/CT compared to MIBI SPECT/CT for preoperative PTA localization in patients with PHPT. Both FCH PET/CT and MIBI SPECT/CT procedures are considered safe, non-invasive, and exposure to ionizing radiation in both tests is low. However, FCH PET/CT is about three to four times costlier for society than MIBI SPECT/CT.

Evaluating a novel imaging technology by means of a phase III randomized controlled trial makes it possible to estimate whether the new technique really makes a clinical difference. To evaluate the impact of FCH PET/CT as a first-line imaging modality on clinical outcomes and social costs compared to the reference standard test MIBI SPECT/CT, we propose an open diagnostic intervention randomized phase III trial. To our knowledge, this is the first prospective randomized trial aiming to compare two diagnostic strategies for first-line preoperative PTA localization. Such a design is in line with the recommendations on the methods of evaluating diagnostic techniques [17]. In this trial, two diagnostic strategies will be compared in a 1:1 fashion (Fig. 1). The result of the diagnostic test is directly coupled with therapy.

To justify its cost, FCH PET/CT-guided parathyroid surgery should lead to improved patient management, resulting in improved cure rates and a reduction in BCEs and surgical complications. In the phase II APACH1 study [12], we showed that second-line FCH PET/CT allowed for cure in 88% of patients with negative or inconclusive MIBI SPECT/CT. BCE could be avoided in 75% of patients. Surgical complication rates were low. We therefore hypothesize that upfront FCH PET/CT will improve patient care in PHPT and that the reduction in clinical costs will compensate for the increase in imaging costs.

### Trial status

Patient enrolment is underway.

## Data Availability

Not applicable.

## Abbreviations

BCE: Bilateral cervical exploration
CT: Computed tomography
FCH: F18-choline
MIBI: Tc99m-sestaMIBI
MIP: Minimally invasive parathyroidectomy
PET/CT: Positron emission tomography/computed tomography
PHPT: Primary hyperparathyroidism
PTA: Parathyroid adenoma
PTH: Parathyroid hormone
SPECT/CT: Single photon emission computed tomography/computed tomography
SPIRIT: Standard protocol items, namely recommendations for interventional trials
